# Supplementary material for: Spin disorder control of topological spin texture
Source: Nat Commun. 2024 May 7;15:3828. doi: 10.1038/s41467-024-47715-5 (PMC11076609; doi:10.1038/s41467-024-47715-5)
Supplement: Supplementary file 1 — Supplementary Information [file 41467_2024_47715_MOESM1_ESM.pdf]

Supplementary information for:

Spin disorder control of topological spin texture

Hongrui Zhang *et al.*

Supplementary Note 1 Discussion of the origin of magnetic phase separation.
Supplementary Note 2 Discussion of domain phase separation.
Supplementary Fig. 1 Single-crystal XRD for bulk Fe_3GaTe_2.
Supplementary Fig. 2 Visualization of Fe^{int} intensities in atomic-resolution iDPC-STEM image of Fe_3GaTe_2.
Supplementary Fig. 3 Magnetic properties of bulk Fe_3GaTe_2.
Supplementary Fig. 4. Thickness-dependent anomalous Hall curves at various temperatures of Fe_3GaTe_2.
Supplementary Fig. 5 X-ray magnetic circular dichroism measurements of bulk Fe_3GaTe_2.
Supplementary Fig. 6 Field-induced magnetic phase transition and exchange bias at low Temperature.
Supplementary Fig. 7 Anomalous Hall curves at various temperatures after ZFC.
Supplementary Fig. 8 Calculation of magnetic exchange coupling of Fe_3GaTe_2 with Fe^{int}.
Supplementary Fig. 9 Raman mapping.
Supplementary Fig. 10 SHG mapping.
Supplementary Fig. 11 STM image.
Supplementary Fig. 12 Comparison of magnetic-phase separation under two identical thermal processes.
Supplementary Fig. 13 The schematic energy landscape of the field- and temperature-induced phase transition.
Supplementary Fig. 14 Monte Carlo simulations.
Supplementary Fig. 15 Thickness dependence of magnetic domain.
Supplementary Fig. 16 Micromagnetic simulation.
Supplementary Fig. 17 Observation of disordered spins in the Fe_3GeTe_2 with Fe^{int}.
Supplementary Fig. 18 The schematic of the formation process of zero-field skyrmion lattice.
Supplementary Fig. 19 Lorentz TEM contrast of Fe_3GaTe_2 flakes at room temperature.
Supplementary Fig. 20 Magnetic field-induced magnetic phase transition.
Supplementary Fig. 21 Current-induced skyrmion motion at room temperature.
Supplementary Fig. 22 DMI discussion of Fe_3GaTe_2 with/without Fe^{int}.
Supplementary Table 1. Calculation of magnetic exchange coupling of Fe_3GaTe_2 with Fe^{int}.
Supplementary Table 2. DMI discussion of Fe_3GaTe_2 with/without Fe^{int}.

Supplementary Note 1 Discussion of the origin of magnetic phase separation.

To better understand the origin of the magnetic-phase separation in Fe_3GaTe_2, we first rule out the existence of chemical and structural inhomogeneities at the same length scale. Raman, optical second harmonic generation mapping with sub-micrometer spatial resolution and scanning tunneling microscope with atomic spatial resolution validates the structural homogeneity in Fe_3GaTe_2 (Supplementary Figs. 9-11). In addition, the Ferri- and Ferro-phase regions can be easily altered by performing thermal treatments (*e.g.*, heating to 400 K and cooling back to room temperature), as evidenced in the two MFM images acquired at the same position at room temperature before and after thermal cycling (Supplementary Fig. 12). Furthermore, the Ferri-phase can transition into a ferromagnetic state by changing the temperature or applying magnetic fields. (Supplementary Fig. 13) Thus, the magnetic phase coexistence is not simply due to nanoscale chemical phase separation or structural inhomogeneity. Instead, it arises from the competition of different magnetic states with ground state energies that are relatively close to each other, assisted by thermal fluctuations.

Supplementary Note 2 Discussion of domain phase separation.

In a system with perpendicular magnetizations, the long-range magnetic dipole-dipole interaction favors antiparallelly aligned magnetizations of neighbor magnetic domains. In labyrinthine domains, which can be regarded as curling stripes, there are more “neighbors” of antiparallelly aligned perpendicular magnetizations between the neighbor stripes. Therefore, the magnetic dipole-dipole interaction energy of labyrinthine domains is lower than that of straight parallel stripe domains. On the other hand, labyrinthine domains have longer and curling domain walls than straight parallel stripe domains, which results in higher domain wall energy. In the Ferri-phase regions, antiferromagnetic interlayer coupling results in smaller net magnetization of stripe domains, which attenuates the dipole-dipole interaction. Therefore, the domain wall energy dominates, and it favors the formation of long, straight, parallel stripe domains. Defects can also induce random short-range magnetic interactions, leading to stripe dislocations and labyrinthine domains as the temperature decreases from T_c, which explains the experimental observation in the Ferro-phase regions.

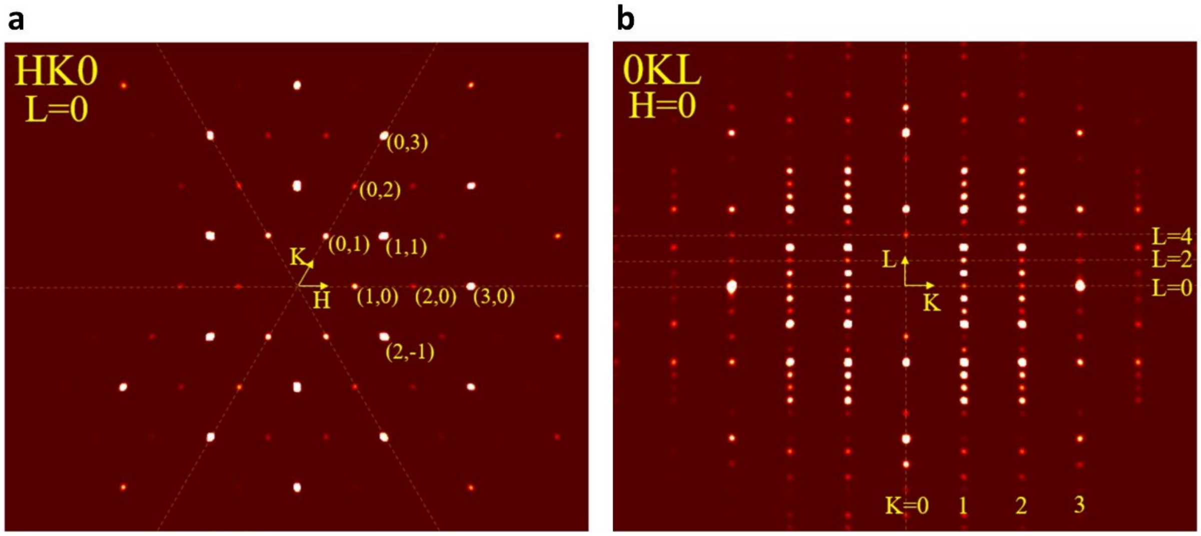

Supplementary Fig. 1 Single-crystal XRD for bulk Fe_3GaTe_2. We performed the single-crystal XRD measurement on the Fe_3GaTe_2 with 8.5% Fe^{int}. The EDS measurement obtains the element ratio of the iron, gallium, and tellurium elements as 3.09: 0.96: 2. Single-crystal XRD patterns of the HK0 and 0KL planes are shown in **a**, **b**. The sharp diffraction spots and the missing streak signals indicate the high quality of the crystal. After the refinement of the data, the obtained lattice constants are a = b = 4.080 Å, and c = 16.138 Å. The site occupancy of the Fe^{int} is ~ 8.5%, coincidentally equal to the excess of the stoichiometry.

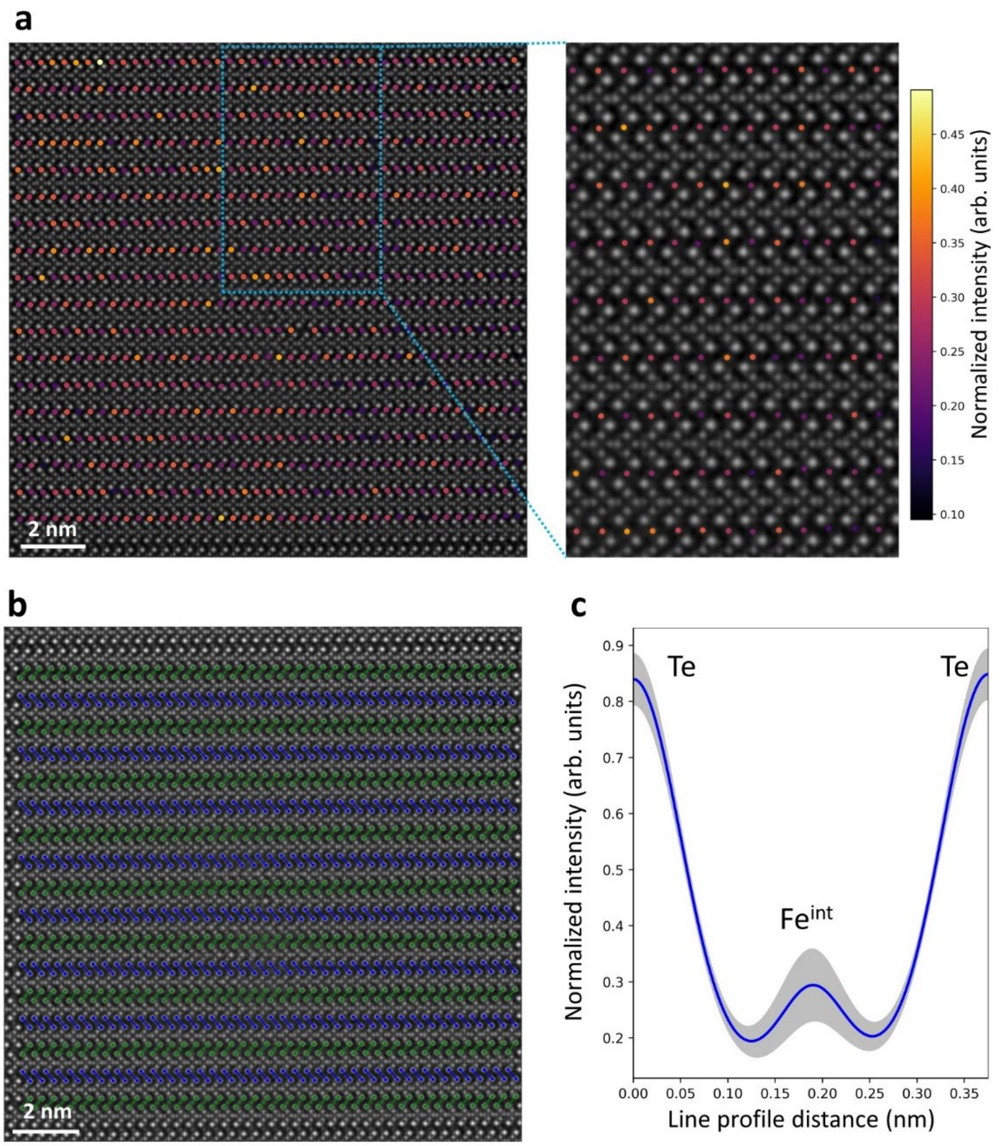

Supplementary Fig. 2 Visualization of Fe^{int} intensities in atomic-resolution iDPC-STEM image of Fe_3GaTe_2. **a**, Using 2D Gaussian peak fitting of the atomic-resolution cross-section iDPC-STEM image of Fe_3GaTe_2 with 8.5% Fe^{int}, the intensities of Fe^{int} can be obtained and directly visualized, confirming the nanometer-scale inhomogeneous distribution of the intercalated Fe atoms. **b,** Atomic-resolution cross-section iDPC-STEM image of Fe_3GaTe_2 with 8.5% Fe^{int}, in which the Te-Fe^{int}-Te atomic planes were indicated as blue and green peaks. **c**, The line profiles can be obtained from the identified Te-Fe^{int}-Te peaks, in which the blue and gray curves indicate the average and standard deviations over 336-line profiles, respectively. The low and large variation of intensity further confirms that the Fe^{int} atoms partially and randomly occupy the octahedral intercalated sites.

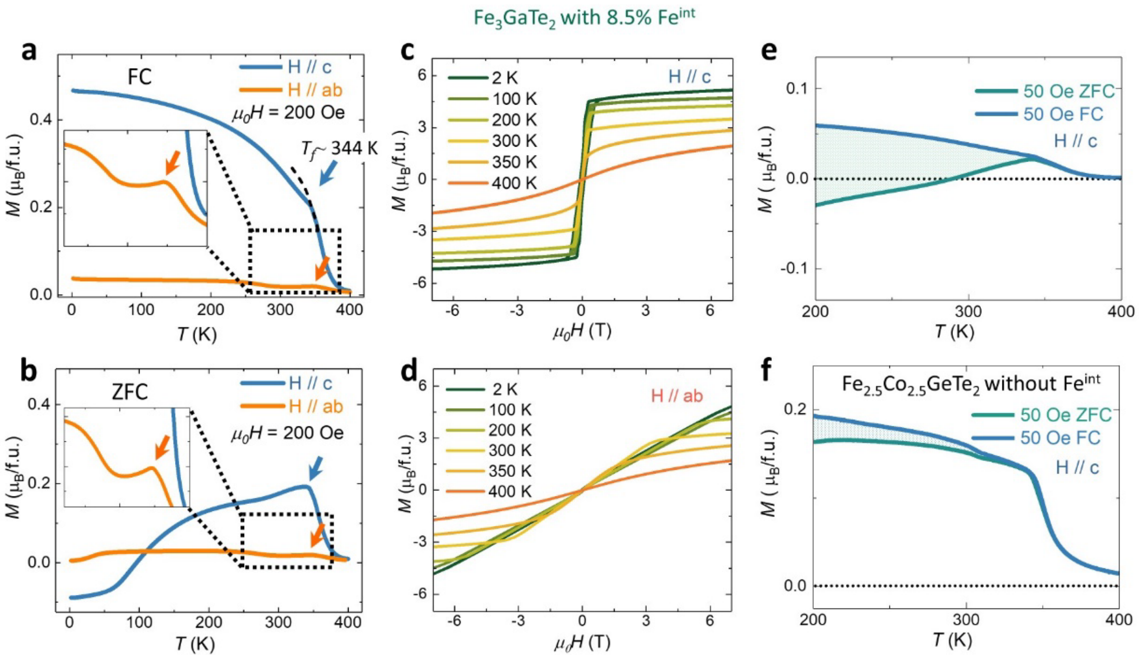

Supplementary Fig. 3 Magnetic properties of bulk Fe_3GaTe_2. The temperature-dependent magnetization (M-T) was measured under both the out-of-plane (blue) and in-plane (orange) magnetic fields of 200 Oe after field cooling (FC) (**a**) and zero-field cooling (ZFC) (**b**). The magnetization below frozen temperature (T_f) decreases in all M-T curves, indicating the emergence of the antiferromagnetic phase instead of a spin reorientation. Isothermal magnetization curves of a bulk crystal were measured at various temperatures under both out-of-plane (**c**) and in-plane (**d**) magnetic fields. At 2 K, the magnetization is not saturated until the in-plane field is above 7 T, indicating an easy axis along the *c*-axis of the crystal. The in-plane saturated field is ~ 3.4 T at 300 K, with a calculated magnetic anisotropy energy ($K_u = M_s H_k/2$, where M_s is the saturation magnetization, H_k the anisotropy field) of ~ 4.0 $\times 10^5$ J/m^3. We compared magnetic properties between Fe_3GaTe_2 with Fe^{int} and $Fe_{2.5}Co_{2.5}GeTe_2$ without Fe^{int} in Fig. **e**,**f**. The bifurcation between ZFC and FC MT curves is more pronounced in Fe_3GaTe_2 with 8.5% Fe^{int} compared to $Fe_{2.5}Co_{2.5}GeTe_2$. This suggests that the Fe^{int} induces more random spins in the Fe_3GaTe_2 with Fe^{int}.

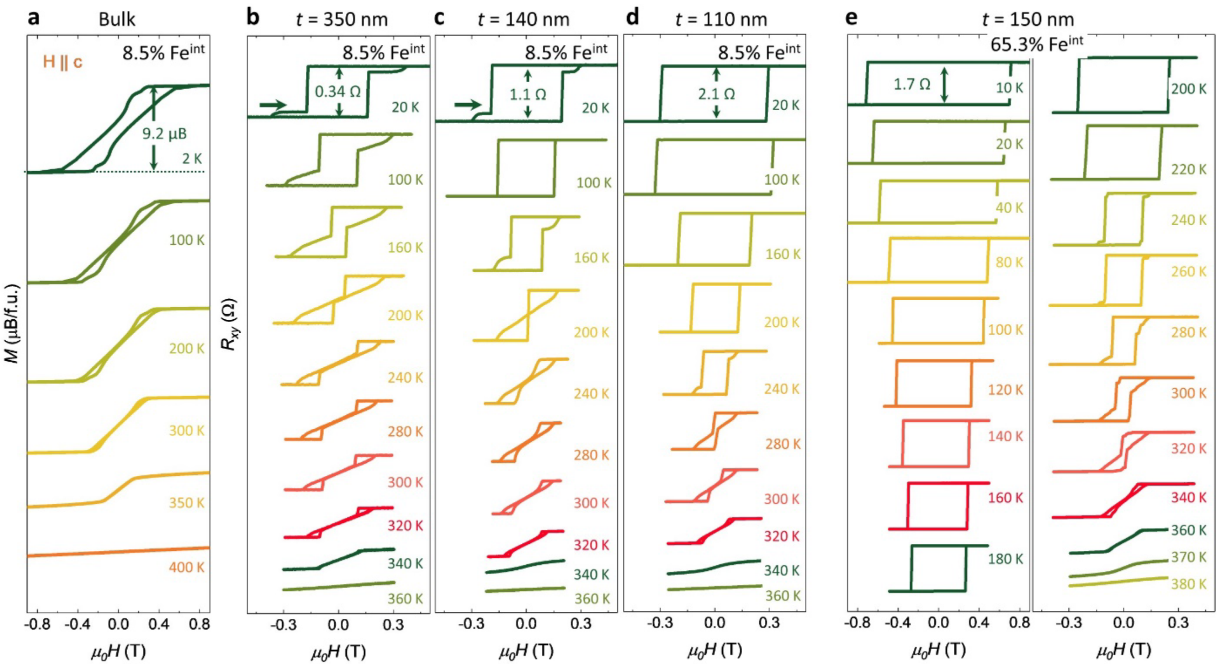

Supplementary Fig. 4. Thickness-dependent anomalous Hall curves at various temperatures of Fe_3GaTe_2. a, Isothermal magnetization curves of a bulk crystal at various temperatures. **b-e**, Anomalous Hall curves of Fe_3GaTe_2 nanoflakes with 8.5% Fe^{int} (t = 350 nm, 140 nm, and 110 nm) and Fe_3GaTe_2 nanoflakes with 65.3% Fe^{int} (t = 150 nm) obtained at different temperatures. A kink was observed at high magnetic fields in the anomalous Hall curves at low temperatures in Figs. **b** and **c**, marked by the arrows, attributed to the spin-flop transition of the interlayer antiferromagnetic phase. Since the antiferromagnetic domain size decreases as the scale along the *c*-direction decreases, it is difficult to stabilize a long-range antiferromagnetic order in thin nanoflakes, even at low temperatures. Thus, for t = 110 nm, the anomalous Hall curves display standard square shapes without a spin-flop transition at low temperatures in Fig. **d**. No visible spin-flop transition occurs at low temperatures in Fe_3GaTe_2 nanoflakes with 65.3% Fe^{int} (Fig. **e**), attributed to the relatively high energy barrier between the antiferromagnetic and ferromagnetic state at high Fe^{int} concentrations. Hence, the antiferromagnetic state is readily stabilized at low temperatures in thick Fe_3GaTe_2 nanoflakes with lower Fe^{int} concentrations.

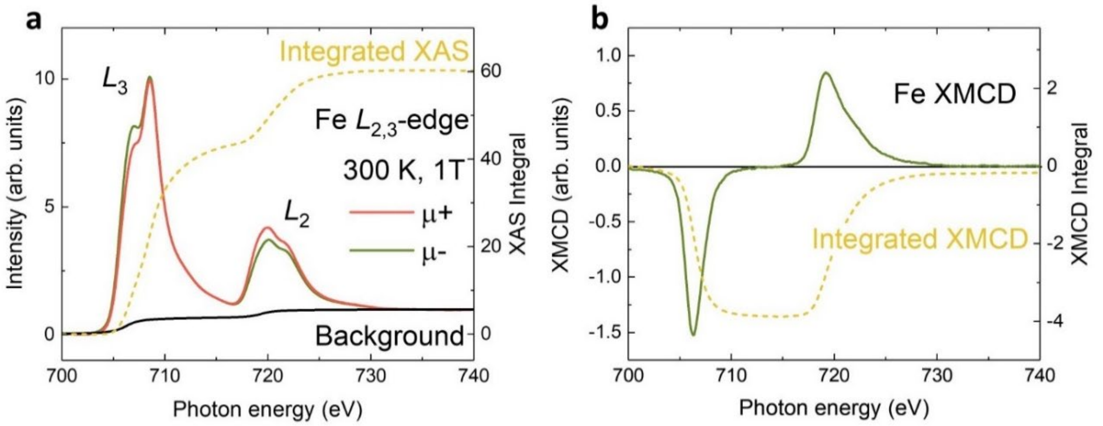

Supplementary Fig. 5 X-ray magnetic circular dichroism measurements of bulk Fe_3GaTe_2. The Fe $L_{2,3}$-edge X-ray absorption spectra (XAS, **a**) and X-ray magnetic circular dichroism (XMCD, **b**) were measured at 300 K under 1 T, confirming the ferromagnetic ground state of Fe_3GaTe_2 above room temperature. Before the measurement, we cleaved the crystal to remove the surface oxidized layer and eliminate its potential impact on the results. Using the sum rules[1], the spin (m_{spin}) and orbital (m_{orb}) moment were evaluated, which yielded m_{spin} = 0.924 μB/Fe and m_{orb} = 0.020 μB/Fe, respectively. The estimated total moment is 0.944 μB/Fe, which is close to the value from the macroscopic magnetometry. The spin and orbital moments are parallel because the Fe atoms in Fe_3GaTe_2 have more than half-filled electron shells.

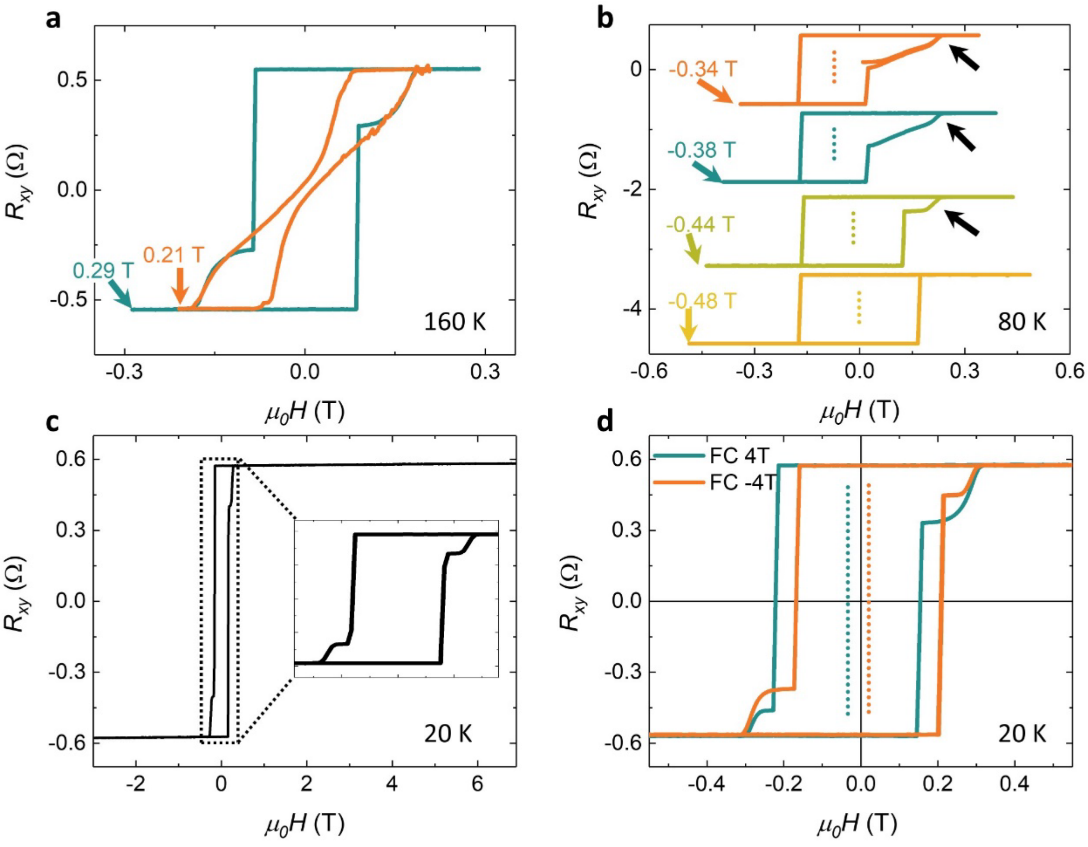

Supplementary Fig. 6 Field-induced magnetic phase transition and exchange bias at low Temperature. a, The anomalous Hall curves for the 140 nm thick Fe_3GaTe_2 nanoflake with 8.5% Fe^{int} were measured at 160 K under the two different scanning ranges of the magnetic field. Note that all of the applying magnetic fields were larger than the saturation magnetic field (~0.19 T). When the field was swept back and forth between -0.21 T and 0.21 T, the anomalous Hall curve showed a sheared out-of-plane hysteresis loop, indicating a multidomain state. We increased the scanning field up to ± 0.29 T; interestingly, the curve displays distinct hysteresis loops consisting of two loops with different shapes. Similar behaviors were also observed at 80 K in Fig. **b**. As the range of the scanning loops increases, the loops finally transition from a multidomain state into a standard square-shaped hysteresis loop. This indicates that even when the applied magnetic field slightly exceeds the saturation field, there are still frozen antiferromagnetically coupled spins in the system that act as nucleation centers, leading to the domain remaining in a multidomain state. As the applied field further increases, the random spins will gradually rotate along the external field, leading to a single-domain state. These further confirmed that the energies of the antiferromagnetic and ferromagnetic states are close, and the magnetic field can align the frozen

antiferromagnetic spins. **c**, The two-step loop is still there at 20 K, even if the applying field reaches up to 7 T, indicating that the magnetic field hardly aligns the frozen antiferromagnetic spins at low temperatures. **d**, The exchange bias behavior occurs at 20 K after positive and negative magnetic field cooling, further confirming the existence of the antiferromagnetic phase.

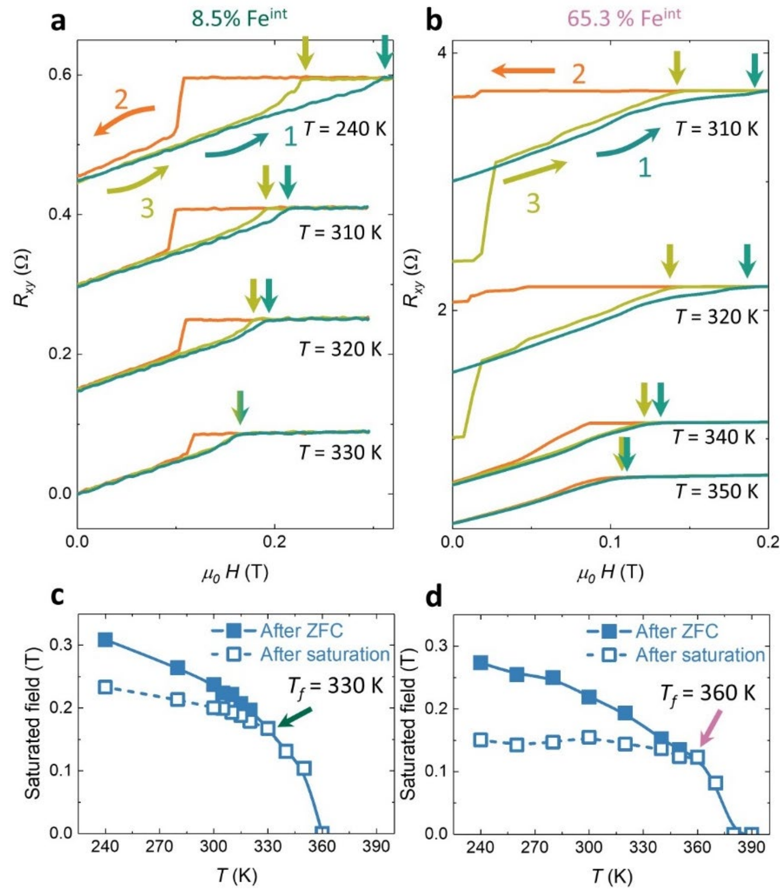

Supplementary Fig. 7 Anomalous Hall curves at various temperatures after ZFC. a, b Anomalous Hall curves were measured at different temperatures after ZFC. The sweep field process followed the sequence '1' ~ '2' ~ '3'. When the temperature is below T_f, the initial magnetization curves ('1') are located outside of the primary hysteresis loops ('3'). Once T > T_f, the initial magnetization curves ('1') overlap with the primary hysteresis loops ('3'). Finally, we summarized the temperature dependence of the saturated field after both ZFC and magnetization saturation in Figs. **c**, **d**, indicating that the T_f is ~ 330 K and ~ 360 K in Fe_3GaTe_2 with 8.5% Fe^{int} and 65.3% Fe^{int}, respectively.

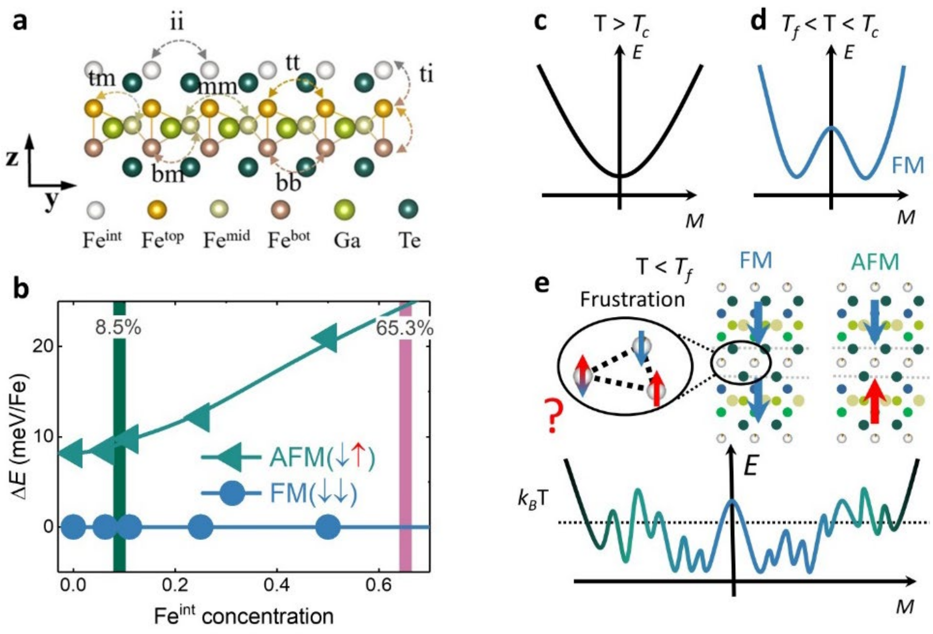

Supplementary Fig. 8 Calculation of magnetic exchange coupling of Fe_3GaTe_2 with Fe^{int}. a, Side view of a monolayer Fe_3GaTe_2 with intercalated iron atoms. The intercalated, top, middle, and bottom Fe ions are denoted as Fe^{int}, Fe^{top}, Fe^{mid}, and Fe^{bot}, respectively. Note that Fe^{top} and Fe^{bot} are symmetrically equivalent in Fe_3GaTe_2 but not in Fe_3GaTe_2 with Fe^{int}. The considered exchange paths are shown by the double arrows. For the Fe_3GaTe_2, the first-nearest neighbor (Fe^{top}-Fe^{bot}), the second-nearest neighbor (Fe^{top}-Fe^{mid}, or equivalently Fe^{bot}-Fe^{mid}), and the third-nearest neighbor (Fe^{top}-Fe^{top}, or equivalently Fe^{bot}-Fe^{bot}) all yield strong ferromagnetism (Table 1). The third-nearest neighbor (Fe^{mid}-Fe^{mid}) favors weak antiferromagnetism with J^{mm} = 4.3 meV. In contrast, by introducing the intercalated atoms Fe^{int}, the exchange coupling between the Fe_3GaTe_2 and intercalated atom (Fe^{top}-Fe^{int}) is strongly ferromagnetic with J^{ti} = -187.1 meV. The ferromagnetic J^{tb} rapidly decreases compared with that of the freestanding Fe_3GaTe_2. This can be understood as the enhancement of exchange coupling between Fe^{int} and $Fe^{top,}$ resulting in the reduction of adjacent interactions of Fe_3GaTe_2 itself. Note that the J^{tm} and J^{bm}, as well as the J^{tt} and J^{bb}, are non-equivalent in Fe_3GaTe_2 with Fe^{int} since both the overlap integral and local symmetry are strongly altered by the intercalation effect. Especially, the antiferromagnetic J^{mm} increases obviously, compared with that of Fe_3GaTe_2. Besides, the introduced intercalated iron atoms (Fe^{int}-Fe^{int}) favor strong antiferromagnetism with J^{ii} = 17.3 meV. Such interactions yield geometric frustration (antiferromagnetism in triangular sublattice) and compete with the aforementioned ferromagnetic ones, which weakens ferromagnetism and helps stabilize non-

collinear states. Thus, the low Fe^{int} concentration favors a spin glass state; with the increase in Fe^{int} concentration, a spin-frustrated state can be stabilized. **b,** The relative ground state energy of the antiferromagnetic state with respect to the fully ferromagnetic state versus the Fe^{int} concentration in $Fe_{3+x}GaTe_2$. The magnetic ground state of $Fe_{3+x}GaTe_2$ is always an interlayer ferromagnetic coupling. The barrier energy between ferromagnetic and antiferromagnetic are close in Fe_3GaTe_2 without Fe^{int} and increase as the Fe^{int} concentration. Thus, the magnetic state of $Fe_{3+x}GaTe_2$ after ZFC can be depicted as the schematic of the energy landscape in Fig. **c**-**e**. At T < T_f, e.g., at room temperature, the magnetic state of the system manifests the existence of antiferromagnetic couplings within a ferromagnetic background (Fig. **e**).

Supplementary Table 1. Magnetic parameters (in meV) of Fe_3GaTe_2 (FGT) and Fe_3GaTe_2 with full intercalated Fe^{int} (FGTF) MLs under different exchange paths marked in **Supplementary Fig. 8**. The negative and positive values represent the ferromagnetic and antiferromagnetic exchange interactions, respectively.

Exchange paths	tb	tm	bm	mm	tt	bb	ii	ti
**FGT**	-81.4	-61.8	-61.8	4.3	-8.6	-8.6		
**FGTF**	-11.1	-51.2	-31.8	7.93	-10.4	-9.5	17.3	-187.7

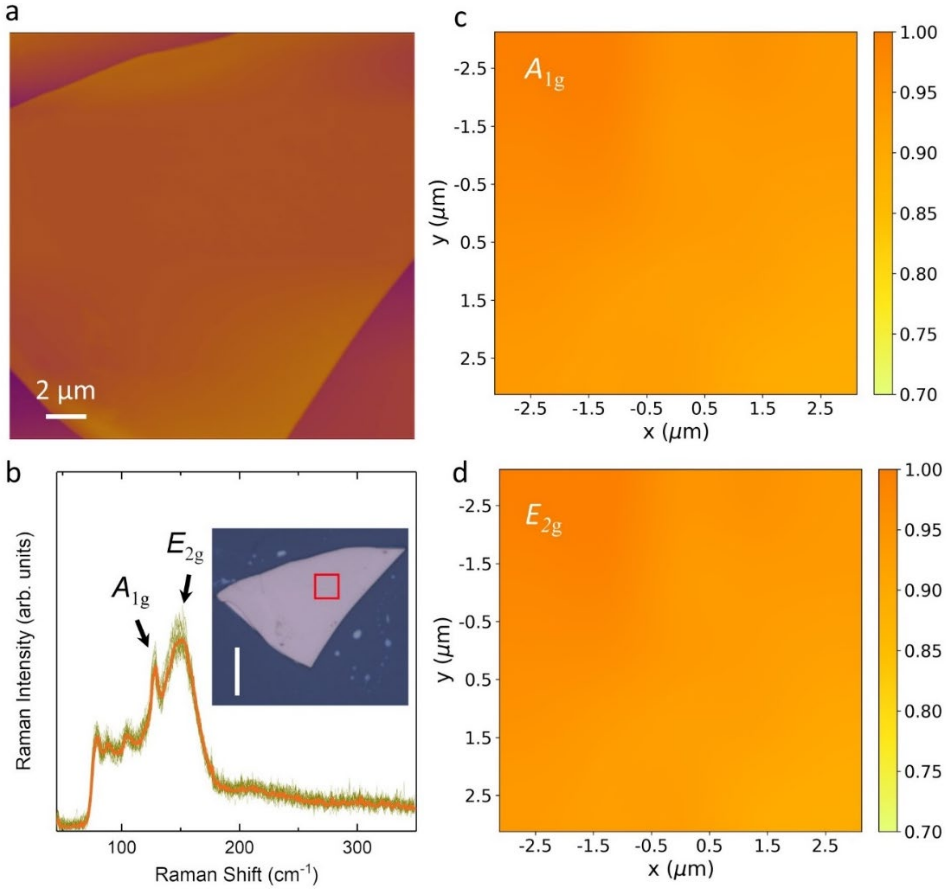

Supplementary Fig. 9 Raman mapping. The Raman measurements were conducted using a Horiba Jobin Yvon LabRAM ARAMIS confocal Raman microscope under 532nm excitation. The laser was focused to a spot around 1 μm^2 through an Olympus 100x objective lens. The polarizer and analyzer were set to a parallel configuration. Fe_3GaTe_2 samples were mounted on an automated XY piezo stage to achieve spatial mapping. **a,** The atomic force microscopy image of the Fe_3GaTe_2 with 8.5% Fe^{int} nanoflake for Raman spectrum measurement. The nanoflake thickness is ~136 nm, obtained by the line profile of the atomic force microscopy image. **b**, Inset: the optical image of the flake. The scale bar is 10 μm. Raman spectra of the Fe_3GaTe_2 nanoflakes were acquired with a scanning region of 5 × 5 μm^2, as indicated by the red box in the inset figure. The laser spots utilized for Raman measurements are smaller than 1 μm in diameter. The Raman peak profiles of Fe_3GaTe_2 8.5% Fe^{int} in Fig. **b** are identified at 128.9 cm^{-1} and 150.4 cm^{-1}, which can be attributed to the A_{1g} and E_{2g} phonon modes, respectively. No measurable shift in the peak position of the Raman profiles is detected within the whole region. Raman spectrum mapping using both the A_{1g} (**c**) and E_{2g} (**d**) mode on the Fe_3GaTe_2 nanoflake indicates that Fe^{int} atoms have an inhomogeneous distribution.

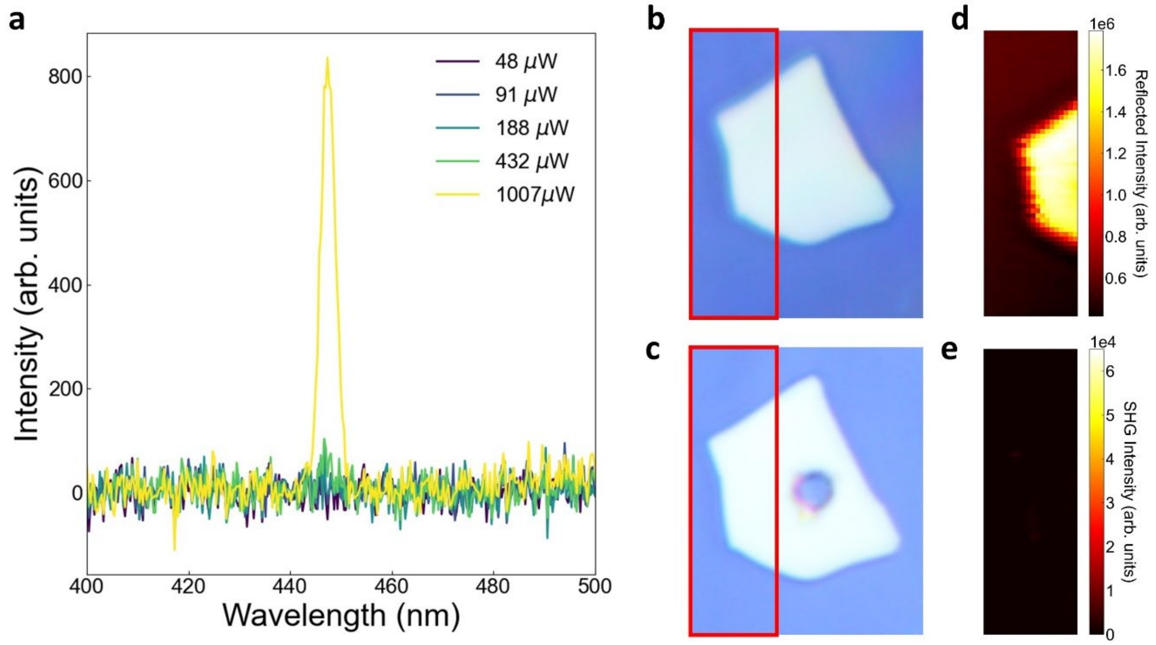

Supplementary Fig. 10 SHG mapping. SHG and Reflection measurements were performed using a Ti: Sapphire oscillator (Chameleon Ultra, Coherent) with ~100 fs pulse widths and a center wavelength of 900 nm. This was done in a normal-incidence reflection geometry. The light was focused using a 100x (NA=0.95) objective. For reflection measurements, the laser was sent to the objective, and the signal was reflected back through a 50:50 beam-splitter, whereas for SHG a 720 nm short pass dichroic beam-splitter was used with additional filtering of the backscattered signal using a 580 nm short pass filter. The signal was collected using a spectrometer (Spectra Pro 500i, Acton Research Instruments) with a charge-coupled camera (Andor iXon CCD). **a,** Power-dependent second harmonic generation (SHG) spectrum was measured for an as-grown Fe_3GaTe_2 with 8.5% Fe^{int} nanoflake at room temperature. At low laser energy, no measurable SHG single was observed. Once the sample was damaged by the high-energy laser heating, the SHG signal was observed. The optical images of the nanoflake before and after damage are exhibited in **b** and **c**, respectively. The laser reflection image of the nanoflake (**d**) in the red box in Fig. **b** corresponds to the SHG mapping (**e**). No signal is observed in SHG mapping, which indicates that there is no in-plane breaking of inversion symmetry in this system. This also implies no long-range ordering of the Fe^{int} within the vdW gap.

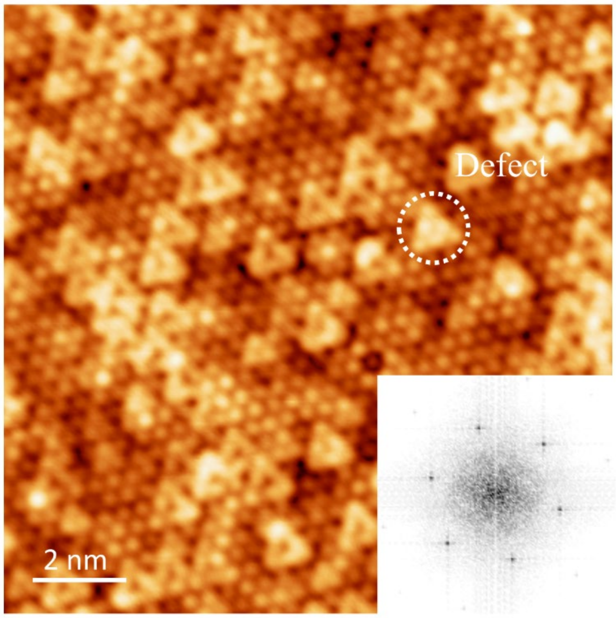

Supplementary Fig. 11 STM image. Single crystals of Fe_3GaTe_2 were cleaved under ultra-high vacuum (UHV) at room temperature. After cleaving, the samples were immediately transferred onto the STM head for measurement. All scanning tunneling microscopy (STM) data were acquired in a commercial Omicron low temperature-STM held at $T = 4.7$ K using a tungsten tip. The tip was prepared on a Cu (111) surface and calibrated against the Cu (111) surface state. All STM images were processed using WSxM software[2]. Scanning tunneling microscope (STM) topographic images of the Fe_3GaTe_2 with 8.5% Fe^{int} surface reveal hexagonal lattices attributed to the uppermost tellurium atoms ($V_{bias} = -10$ mV, $I_t = 1$ nA). The obtained in-plane lattice constant is 4.09 ± 0.07 Å, which is consistent with the single-crystal XRD results. Surface defects of triangular atomic clusters are observed (e.g. white circle above), which are attributed to the presence of Fe^{int} atoms remaining at the cleaved surface. The coverage of the triangular surface defects averaged from 12 areas randomly selected on the sample surface is 10.8% ±7.7%, which is consistent with the results of the single-crystal XRD refinement. Unlike the single-crystal XRD and STEM results, where the Fe^{int} atoms are located at the center of the trigonal tellurium lattice, the surface iron atoms in the STM images tend to align with the tellurium lattice. This could be explained by the reconstruction of surface iron atoms due to the different chemical environments between surface and bulk.

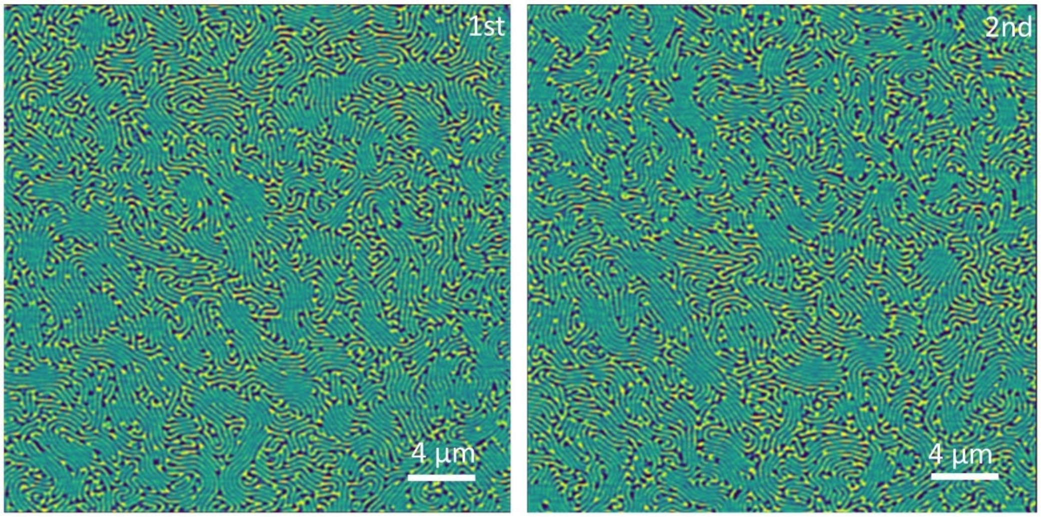

Supplementary Fig. 12 Comparison of magnetic-phase separation under two identical thermal processes. MFM images were captured for the 211-nm-thick Fe_3GaTe_2 nanoflake with 8.5% Fe^{int} at room temperature and zero field after undergoing twice zero-field cooling processes. The regions of magnetic-phase separation for the two images are different, indicating that the two-phase separation does not arise from a chemical or structural phase separation.

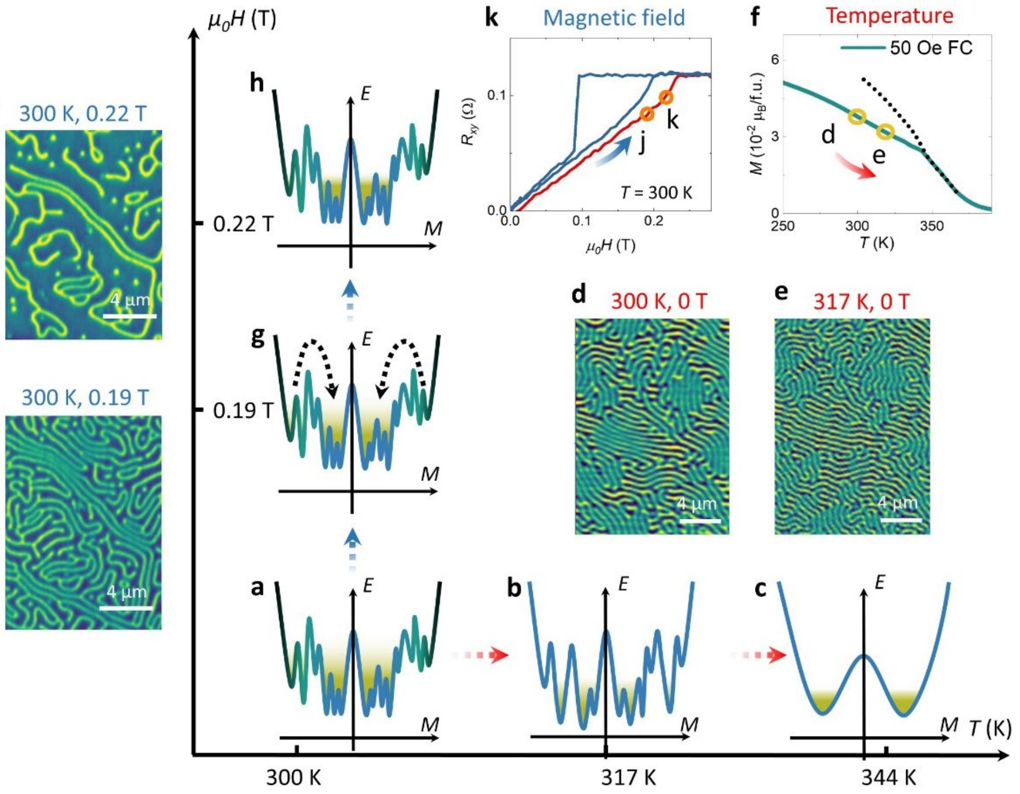

Supplementary Fig. 13 The schematic energy landscape of the field- and temperature-induced phase transition. a, The schematic energy landscape of the Fe_3GaTe_2 with 8.5% Fe^{int} after ZFC. Thermal fluctuation near T_c can overcome the energy barrier between the antiferromagnetic and ferromagnetic states, thus forming a coexistence of antiferromagnetic and ferromagnetic states after cooling down near T_c. When heating the sample (**b**, **c**) or applying a magnetic field (**g**, **h**), the antiferromagnetic state transitions into the ferromagnetic state, as follows the energy landscape. The MFM images of Figs. **d**, **e** are measured at yellow circles indicated in the M-T curve (Fig. **f**). The MFM images of Figs. **j**, **i** are measured at orange circles indicated in the anomalous Hall curve (Fig. **k**). Correspondingly, the contrast of MFM images becomes uniform, as shown in Figs. **e**, **i.**

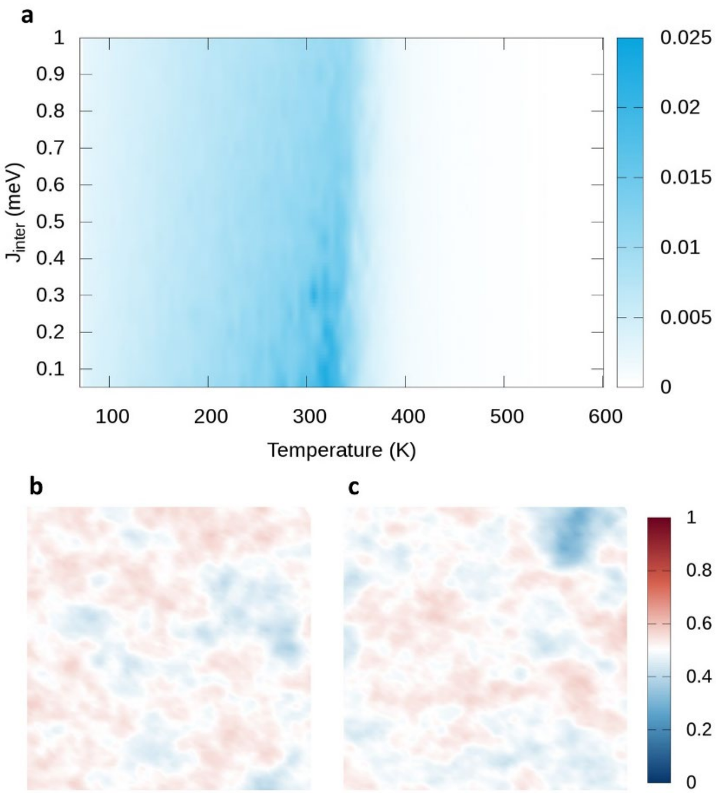

Supplementary Fig. 14 Monte Carlo simulations. a, Simulated phase diagram of magnetic-phase separation as a function of Temperature and J_{inter} (magnetic coupling between adjacent interlayers). We carried out Monte Carlo (MC) simulations for Fe_3GaTe_2 bulk using the calculated magnetic exchange interactions in Tables 1 and 2 and various J_{inter}. The color bar represents Φ. Here, we define the degree of ferromagnetic/antiferromagnetic phase separation as

$$\phi = \sqrt{\phi_{\text{FM}} \phi_{\text{AFM}}}$$

, where Φ_{FM} and Φ_{AFM} are the order parameters of the ferromagnetic and antiferromagnetic phases.

$$\phi_{\text{FM}} = \frac{1}{2N} \sum_{i=1,N} | S_z^i(1) + S_z^i(2) |,$$

$$\phi_{\text{AFM}} = \frac{1}{2N} \sum_{i=1,N} | S_z^i(1) - S_z^i(2) |$$

Where 1 and 2 denote adjacent layer 1 and layer 2, N is the number of spins in each layer, S_z^i is the z component of the spin vector of the site i averaged over 10000 Monte Carlo steps. Then, for the pure phase (ferromagnetic, antiferromagentic, and paramagnetic), $\Phi = 0$. The non-zero Φ only

corresponds to ferromagnetic/antiferromagnetic phase separations. In Panel **a**, the magnetic-phase separation was reproduced below the T_N. To validate this, we further simulate the magnetic ordering at room temperature using J_{inter} = 1 (**b**) and 0.5 meV(**c**). Although the J_{inter} here is relatively small, due to the low barrier between the ferromagnetic and antiferromagnetic phase, it could satisfy a suitable local J_{inter} under the interplay of thermal fluctuations and intercalating. The 100 × 100 × 1 supercells in Figs. **b** and **c**, are adopted in the MC simulation. The red color represents the ferromagnetic phase, and the blue color represents the antiferromagnetic phase. An antiferromagnetic phase can be observed in these two figures, confirming the existence of ferromagnetic/antiferromagnetic phase separation.

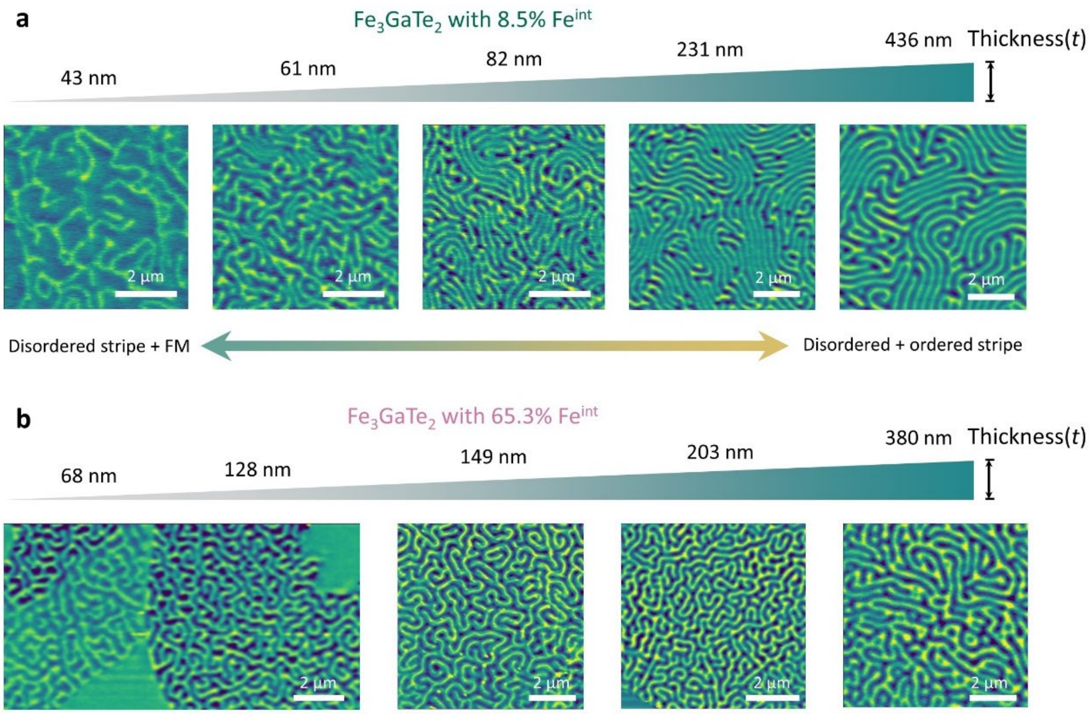

Supplementary Fig. 15 Thickness dependence of magnetic domain. Thickness-dependent MFM images for Fe_3GaTe_2 nanoflakes with 8.5% Fe^{int} (**a**) and 65.3 % Fe^{int} (**b**) at room temperature. The magnetic domain exhibits two phases in the thick Fe_3GaTe_2 nanoflakes with 8.5% Fe^{int}. In the 43 nm thick nanoflake, only dislocations or short domains were observed. All MFM images for Fe_3GaTe_2 nanoflakes with 65.3 % Fe^{int} display the labyrinthine domain.

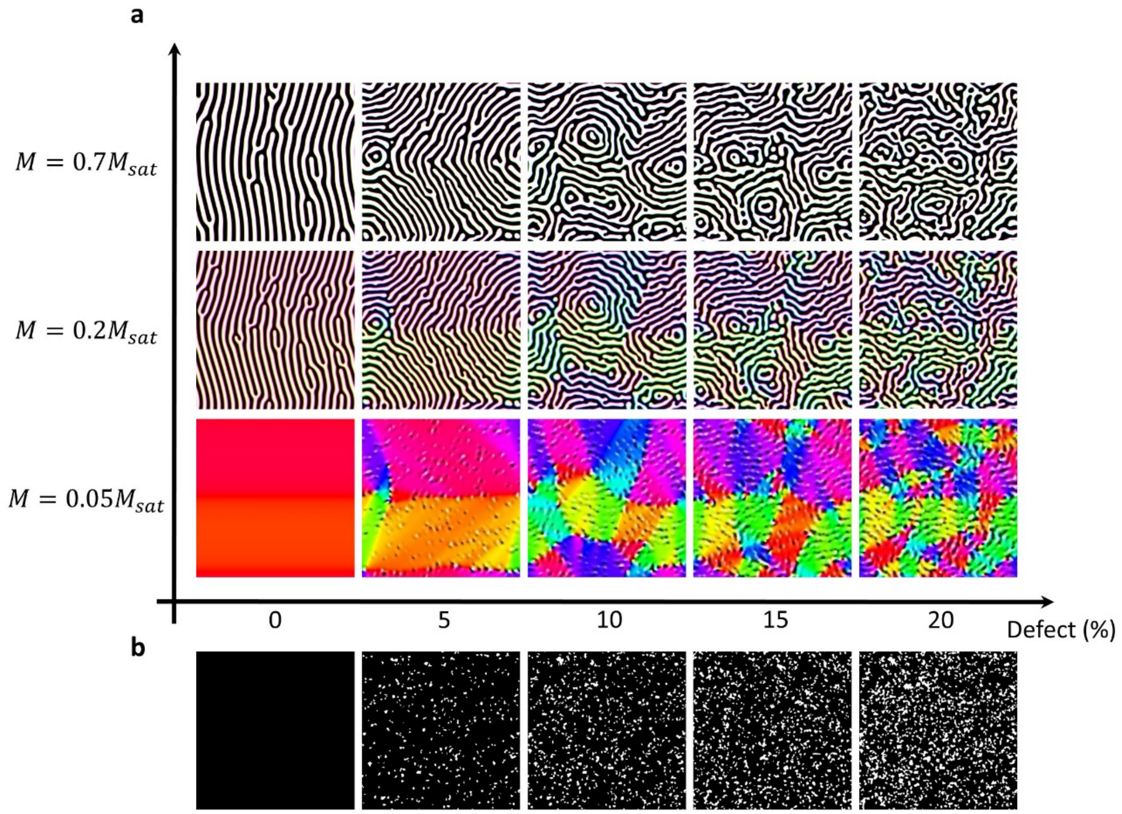

Supplementary Fig. 16 Micromagnetic simulation. a, Evolution of magnetic domains as the saturation magnetization gradually increases when the temperature decreases from around T_c. At each defect level, there is one continuous simulation with the evolution of the magnetic parameters. Every simulation starts from randomized initial magnetization. As the defect density increases, the magnetic domain pattern tends to form a complex labyrinthine domain, closely reproducing the experimental results. The image size is 4.5×4.5 μm^2. **b**, Distribution of defects represented by bright spots in each image, with three times of uniaxial magnetic anisotropy energy compared with defect-free dark regions to represent the local magnetic pinning effect from defects phenomenologically.

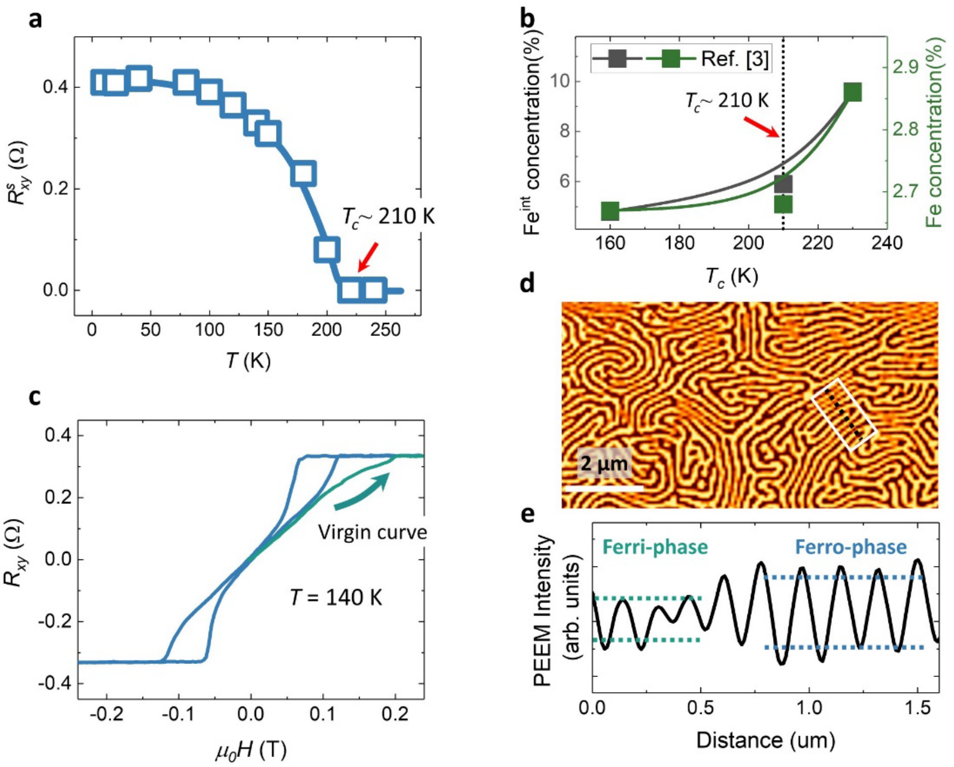

Supplementary Fig. 17 Observation of disordered spins in the Fe_3GeTe_2 with Fe^{int}. a, Temperature dependence of the saturated anomalous Hall resistance (R^{s}_{xy}) for Fe_3GeTe_2 nanoflake with a thickness of 140 nm reveals the T_c of 210 K. In the previous report[3], it was observed that Fe^{int} persistently exists in the vdWs gap of Fe_3GeTe_2, even when the ratio of iron atoms is lower than 3. The Fe/Fe^{int} concentration can tune the T_c of the Fe_3GeTe_2 system, as illustrated in Fig. **b** [3]. According to this relationship, the Fe^{int} concentration in our case can be estimated to be ~ 6.7%. **c**, The anomalous Hall curve was measured at 140 K after ZFC from 300 K. The virgin curve lies outside of the primary hysteresis loops, similar to the Fe_3GaTe_2 with Fe^{int} system, suggesting the existence of random spins. **d**, The XMCD-PEEM image of the Fe_3GeTe_2 with the Fe^{int} system was captured at 110 K after ZFC. We also can observe the two distinct contrast regions. The line profile along the dark dotted line in the white box (Fig. **d)** is displayed in Fig. **e**. The PEEM intensity of the Ferri-phase region, due to the existence of the antiferromagnetic coupled sublayers, is lower than the Ferro-phase region. The domain size of the Ferri-phase (~ 77.6 nm), due to its low stray field, is smaller than the Ferro-phase (91.0 nm).

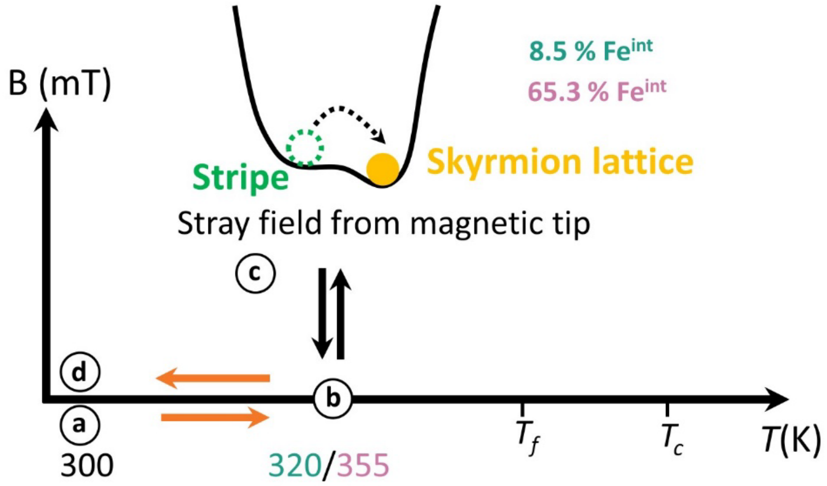

Supplementary Fig. 18 The schematic of the formation process of zero-field skyrmion lattice. First, we performed zero-field cooling on the nanoflakes (**a**). Since the thermal fluctuation helps the stabilization of the skyrmion lattices,[4, 5] (**b**) we slightly heated the nanoflakes. (**c**) Then, the skyrmion lattices were induced after the MFM tips swept the nanoflakes. (**d**) The skyrmion lattices remain when the temperature cools down to room temperature.

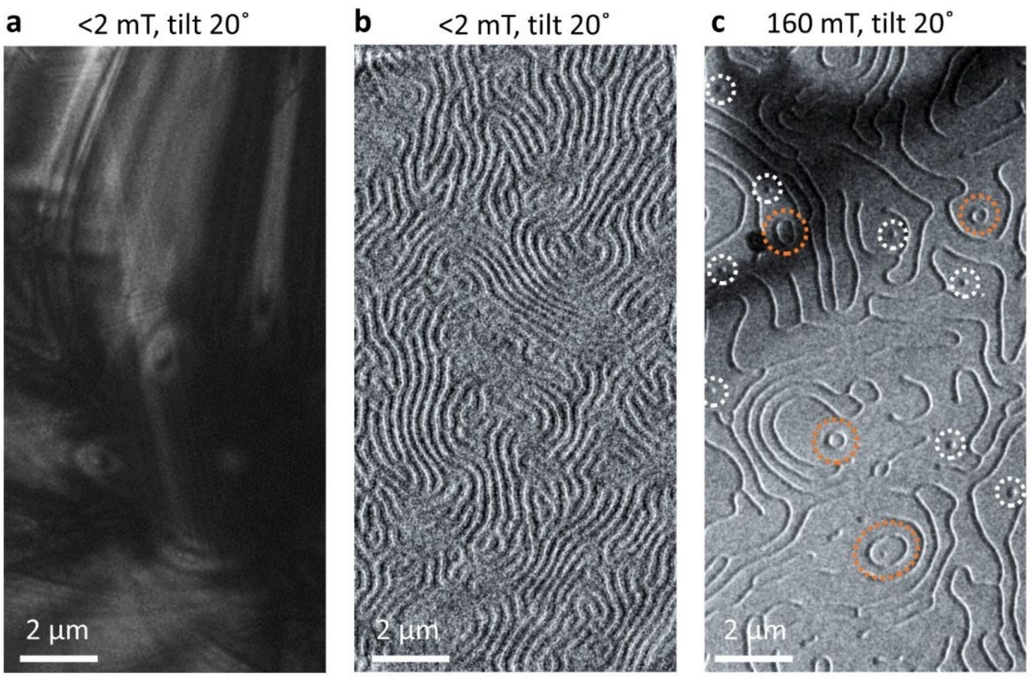

Supplementary Fig. 19 Lorentz TEM contrast of Fe_3GaTe_2 flakes at room temperature. **a,** Lorentz TEM image of a 200 nm thick Fe_3GaTe_2 nanoflake with 8.5% Fe^{int} acquired at room temperature, with a residual field of < 2 mT and a defocus of 3 mm. No magnetic contrast in both the Ferro-phase and Ferri-phase regions was observed without titling the samples. **b**, When the sample is tilted by 20°, the two distinct contrasts emerge in the Lorentz TEM image, which is consistent with the MFM and XMCD-PEEM images. This indicates that magnetic textures are Néel-type and have no measurable titled spins in the Ferri-phase regions. The two images (**a**, **b**) are acquired at nearby regions of the same nanoflake. **c**, Skyrmions (white dotted circles) and skyrmioniums(orange dotted circles) were observed under a magnetic field of 160 mT.

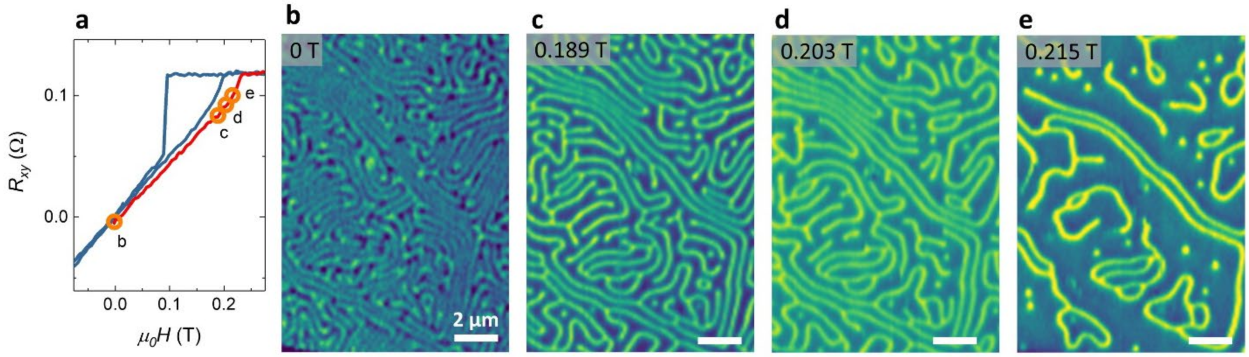

Supplementary Fig. 20 Magnetic field-induced phase transition. a, The anomalous Hall curve of the Fe_3GaTe_2 nanoflake with 8.5% Fe^{int} with a thickness of 350 nm was measured at 300 K after ZFC. The two phases were clearly observed at zero field in Fig. **b**. Then, we gradually increase the magnetic field following the anomalous Hall curves in Fig. **a** (orange circle). **c**, At $\mu_0 H = 0.189$ T, the MFM contrast becomes uniform, indicating the occurrence of phase transition from antiferromagnetic to ferromagnetic state. **d**, **e**, As the magnetic field increases, in the Ferro-phase region, the short stripe domains shrink into bubbles; the ring-shaped dislocation transitions into skyrmionium. After applying a magnetic field larger than the saturation field, all random spins depinning, and the system enters a more stable ferromagnetic state. Then, subsequently removing the field, Fe_3GaTe_2 exhibited a transition in their domain patterns to a labyrinthine domain structure. After this, when a finite magnetic field was applied, the formation of magnetic skyrmion and skyrmionium was not observed in the Fe_3GaTe_2 nanoflake with 8.5% Fe^{int}. Therefore, the formation of topological spin texture in Fe_3GaTe_2 is attributed to the existence of random spins.

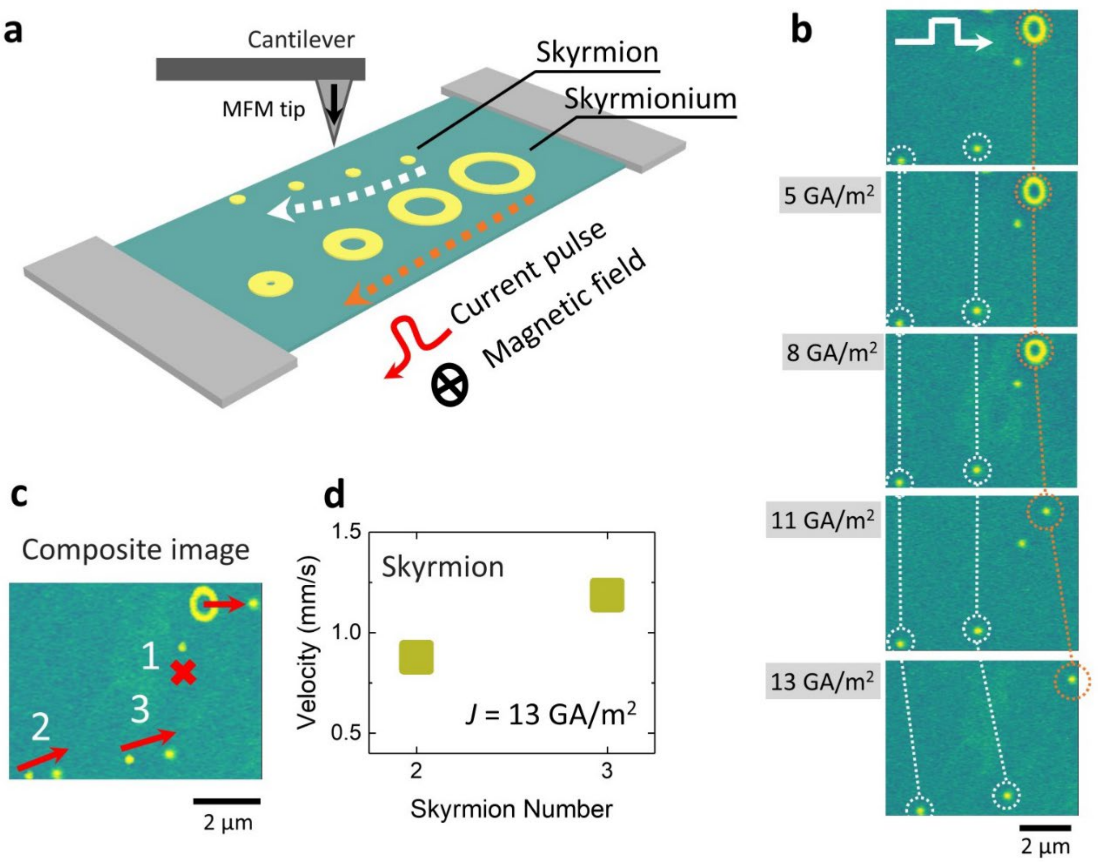

Supplementary Fig. 21 Current-induced skyrmion motion at room temperature. a, The experimental schematic image of current-induced skyrmion motion. An external magnetic field (~ 0.194 T) was applied to a 195 nm thick nanoflake to stabilize the three isolated skyrmions and one skyrmionium in Fig. **b**. Each image was acquired after injecting one current pulse with a duration of 1 ms and various current-density amplitudes. Below the current density of ~ 5 GA/m^2, both skyrmions and skyrmioniums are not motived by pulse current. Slightly above ~ 5 GA/m^2, skyrmioniums start to move and shrink and, finally, collapse into a skyrmion. At j = 13 GA/m^2, the skyrmions start motion or can be annihilated. This indicates that the skyrmionium is more easily moved by pulse current than the skyrmion. The skyrmion motion is not forward along the current direction in Fig. **c**, since the Magnus force moves the skyrmions perpendicular to the current direction, which is the so-called skyrmion Hall effect. The velocity of the skyrmions at room temperature is ~ 1.03 mm/s, at j = 13 GA/m^2 in Fig **d**.

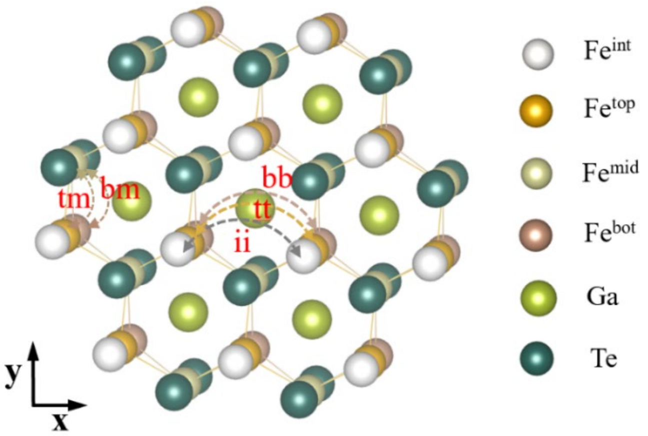

Supplementary Fig. 22 DMI discussion of Fe_3GaTe_2 with/without Fe^{int}. Top view of monolayer Fe_3GaTe_2 with intercalated iron atoms. The DMIs under marked **ii**, **tt**, and **bb** paths are along the y (-y) direction, while they point to the x (-x) direction under the **tm** and **bm** paths. The adopted exchange paths are shown by the double arrows. Here, we take the Fe_3GaTe_2 with fully intercalated Fe^{int} as an example to explore the DMI in this system. Obviously, both the first-nearest neighbor, Fe^{top}-Fe^{int}, Fe^{bot}-Fe^{int}, and Fe^{top}-Fe^{bot}, has mirror symmetries and a 2-fold axis, resulting in a zero DMI between them. The locally broken inversion symmetry of Fe^{top}-Fe^{mid} (Fe^{bot}-Fe^{mid}) yields a local in-plane DMI perpendicular to itself that stabilizes Néel-type skyrmions (Table 2). Similarly, Fe^{top}-Fe^{top} (Fe^{bot}-Fe^{bot}) exhibits a local in-plane DMI as a result of a mirror perpendicular to the Fe^{top}-Fe^{top} bond. Furthermore, the intercalated Fe^{int} may also introduce a new local DMI. Although these local DMIs cancel with each other, the net DMI vanishes. However, random local DMIs induced by disordered Fe^{int} can be considered to stabilize the various topological spin textures.

Supplementary Table 2. The DMI parameters (in meV) of Fe_3GaTe_2 (FGT) and Fe_3GaTe_2 with full intercalated Fe^{int} (FGTF) MLs under different exchange paths marked in Supplementary Fig. 22. The negative and positive values represent the opposite direction.

Exchange paths	tm	bm	mm	tt	bb	ii
**FGT**	2.48	-2.48	0	-2.03	2.03	
**FGTF**	-0.63	-1.23	0.26	0.66	2.21	0.71

Supplementary References

[1] C. T. Chen, *et al.* Experimental Confirmation of the X-Ray Magnetic Circular Dichroism Sum Rules for Iron and Cobalt Phys. Rev. Lett. 75, 152 (1995).

[2] Horcas, I. *et al.* WSXM: A software for scanning probe microscopy and a tool for nanotechnology. *Rev. Sci. Instrum.* **78**, (2007).

[3] Wu, Y. *et al.* Fe-Intercalation Dominated Ferromagnetism of van der Waals Fe_3GeTe_2. Adv. Mater. 35, 2302568, (2023)

[4] S. Mühlbauer, *et al.* Skyrmion Lattice in a Chiral Magnet. Science 323,915-919 (2009).

[5] X. Yu, *et al.* Near room-temperature formation of a skyrmion crystal in thin films of the helimagnet FeGe. Nature Mater. 10, 106–109 (2011).
